# Interaction of intrauterine Zika virus exposure on the relationship between body adiposity and dyslipidemia in school-aged children

**DOI:** 10.3389/fped.2026.1675914

**Published:** 2026-03-02

**Authors:** Aline Ribeiro Murta, Mariana de Santis Filgueiras, Cíntia Pereira Donateli, Milena Sales Thomé, Rosângela Minardi Mitre Cotta, Tiago Ricardo Moreira, Hércia Stampini Duarte Martino, Marcela Benevenuto Ferreira, Glauce Dias da Costa

**Affiliations:** 1 Department of Nutrition and Health, Universidade Federal de Viçosa, Viçosa, Minas Gerais, Brazil; 2 Department of Medicine and Nursing, Universidade Federal de Viçosa, Viçosa, Minas Gerais, Brazil

**Keywords:** dyslipidemia, obesity, overweight, thinness, Zika virus

## Abstract

**Introduction/objectives:**

Intrauterine exposure to the Zika virus (ZIKV) has been primarily associated with neurological outcomes, while its potential metabolic and nutritional consequences remain poorly understood. This study aimed to evaluate the association between anthropometric indicators body mass index (BMI)-for-age, waist circumference, and neck circumference and lipid profile alterations in school-aged children born during the ZIKV epidemic.

**Methods:**

This retrospective cohort included 93 children aged 5–9 years (mean 6.5 ± 0.7 years; 58.1% boys) from the Belo Horizonte Region, Brazil. Participants were classified as exposed (59.1%) or unexposed to ZIKV *in utero*. Anthropometric measurements followed standardized protocols and included BMI-for-age, waist circumference, and neck circumference. Lipid profile assessment included total cholesterol, HDL-c, LDL-c, and triglycerides. Cardiovascular risk was estimated using Castelli indices I and II. Behavioral and sociodemographic factors, including screen time, caregiver education, and family income, were also recorded. Associations between anthropometric indicators and lipid outcomes were analyzed using Poisson regression models with robust variance, including interaction terms to assess the modifying effect of ZIKV exposure.

**Results:**

Lipid abnormalities were common: low HDL-c (44.1%), high total cholesterol (33.3%), high LDL-c (26.9%), and high triglycerides (44.1%). Children exposed to ZIKV had a higher prevalence of low HDL-c compared with unexposed peers (54.6% vs. 29.0%; *p* = 0.015). BMI-for-age was inversely associated with low HDL-c (PR 0.87; 95% CI 0.78–0.97) and showed significant interactions with ZIKV exposure for total cholesterol (p interaction = 0.005) and triglycerides (p interaction = 0.008). Waist circumference interacted with ZIKV exposure regarding total cholesterol (*p* = 0.029; PR 1.09; 95% CI 1.03–1.16). Neck circumference was positively associated with total cholesterol, LDL-c, and triglycerides, with stronger associations among ZIKV-exposed children. Castelli Index I was higher in the exposed group (*p* = 0.0389), while Castelli Index II did not differ significantly (*p* = 0.1087).

**Conclusions:**

Intrauterine ZIKV exposure influences the relationship between adiposity and lipid profile in children. Central adiposity measures including waist circumference, neck circumference, and BMI—provide complementary information for early metabolic risk assessment. These findings highlight the importance of longitudinal monitoring of children exposed to ZIKV *in utero* to detect early metabolic alterations and guide preventive interventions.

## 1 Introduction

At the beginning of 2015, the Zika virus (ZIKV) epidemic struck Brazil, posing a major public health challenge due to the limited knowledge among healthcare professionals about the virus's aggressive neurotropism and its severe consequences for affected children and their families (1–3). Many of these children who were exposed to ZIKV *in utero* have now reached early childhood. While several cohort studies have identified associations between ZIKV exposure during pregnancy and adverse outcomes with long-term neurological sequelae (4–6), a substantial knowledge gap remains regarding its impact on growth, body composition, and metabolic health, as well as the need for targeted long-term follow-up in this population (3).

Recent studies have also shown an increasing prevalence of dyslipidemia in children, both in Brazil and worldwide (7–9), with rates in Brazil ranging from 28% to 40% (10, 11). This increase is exacerbated by behaviors such as physical inactivity and unhealthy food consumption, which contribute to alterations in lipid profile, important markers of cardiovascular risk (12, 13).This trend is closely related to excess weight, a well-established risk factor for coronary artery disease, with dyslipidemia serving as a key marker of cardiometabolic risk (9, 14). Dyslipidemia in childhood is of particular concern because atherosclerosis is known to begin early in life and to progress silently over decades, reinforcing the importance of early detection and longitudinal follow-up (15–17). Evidence shows that fatty streaks and early atherosclerotic lesions can appear in the aorta during childhood and adolescence, reflecting the cumulative impact of metabolic disturbances from a young age (18). Importantly, the development of atherosclerosis is multifactorial, influenced not only by modifiable behaviors but also by genetic and familial risk factors, which contribute significantly to its pathogenesis. This reinforces the need for monitoring lipid profiles in childhood, particularly in populations at risk (19).

However, few studies have investigated the relationship between body adiposity and dyslipidemia in children exposed to ZIKV, especially those affected by congenital Zika syndrome (CZS), which includes conditions such as microcephaly, global developmental delay, epilepsy, visual impairment, and hearing loss (20, 21). Clinical observations suggest that some children aged two to four years with CZS may develop early signs of adiposity and metabolic alterations, even in the absence of excess body weight or central adiposity. These findings raise the possibility that intrauterine ZIKV infection may directly impact adipose tissue development and lipid metabolism, extending beyond its well-known neurological effects (3).

Given the current knowledge gap regarding body adiposity and its long-term relationship with dyslipidemias in children exposed to ZIKV, this retrospective cohort study aimed to evaluate the association between anthropometric indicators [body mass index (BMI)-for-age, waist circumference, and neck circumference] and alterations in the lipid profile in Brazilian children born during the ZIKV epidemic. We hypothesized that higher body adiposity would be associated with an increased prevalence of dyslipidemia, and this association would be more pronounced among children with intrauterine ZIKV exposure.

## 2 Materials and methods

### 2.1 Study design

This is a retrospective cohort study including 93 children, evaluated in two groups: (1) those exposed to the ZIKV during pregnancy, with or without microcephaly (Group 1); and (2) those unexposed to the ZIKV during pregnancy and did not have microcephaly (Group 2).

The study was approved by the Human Research Ethics Committee of the Federal University of Viçosa and the Municipal Health Secretariat of Belo Horizonte, Brazil (Approval codes: 2.705.484 and 3.526.111; Approval dates: March 19, 2021, and August 22, 2019). It was conducted in accordance with the Declaration. The Informed consent was obtained from all legal guardians, and all children fully participated in the procedures outlined in the study protocol.

### 2.2 Setting

The study was conducted in the Metropolitan Region of Belo Horizonte, selected for being a Reference Center in Minas Gerais, Brazil, for the management of pregnant women with suspected or confirmed ZIKV infection, as well as their newborns, regardless of the presence of microcephaly.

### 2.3 Participants

Between March and December 2023, children exposed to ZIKV were identified and selected from the Notifiable Diseases Information System (SINAN), and unexposed children were selected from the Live Births Information System (SINASC). Both systems are part of the Brazilian national health surveillance network. Although occasional underreporting may occur, these databases are considered highly reliable due to mandatory reporting and continuous monitoring.

From the initial records (796 confirmed ZIKV exposures and 100 unexposed births), eligible participants were identified from nominal lists provided by the information systems. Participants were contacted by telephone, following the order of these lists, until the previously calculated sample size was reached. The final sample size (*n* = 93) was defined based on a prior calculation performed using G*Power software (version 3.1.9.7), adopting a 5% significance level, 95% statistical power, and the standard deviation of birth weight reported by Soares et al. (22) and Miot (23).

Recruitment was maintained until the estimated number of participants in each exposure group was reached (55 exposed and 38 unexposed), with an attrition rate of 18% due to refusals, incorrect contact information, or loss to follow-up.

An additional power calculation using the risk of low HDL-c among exposed (65.5%) and unexposed (34.4%) children indicated a relative risk of 1.9 and a statistical power of 80.95%.

Participants were contacted by phone, using the numbers available in the databases. Recruitment continued until the target sample size was reached, with an 18% attrition rate due to refusals, incorrect contact information, or loss to follow-up.

Inclusion criteria comprised children born during the 2015–2017 Zika epidemic, residing in Belo Horizonte Metropolitan Region, and monitored by local health services. Pregnant women exposed to ZIKV met the clinical criteria established by the Centers for Disease Control and Prevention (CDC) for probable ZIKV infection, with notification in SINAN and laboratory confirmation by RT-PCR and/or ZIKV-specific IgM serology, or clinical-epidemiological diagnosis confirmed by health services. Children in the unexposed group were identified based on data from SINASC, matched by age and place of birth. Exclusion criteria was maternal infections during pregnancy (syphilis, toxoplasmosis, rubella, cytomegalovirus, or herpes) and health conditions that could affect lipid or nutritional status. Specifically, children with diagnoses of genetic dyslipidemias, chronic kidney or liver disease, hypothyroidism, diabetes, or use of medications known to alter lipid metabolism were excluded. Children with microcephaly resulting from causes other than ZIKV infection were likewise excluded. In addition, for children with microcephaly, medical records and caregiver reports were reviewed to identify possible additional clinical conditions associated with dyslipidemia. None of these children presented genetic dyslipidemias, chronic kidney or liver disease, hypothyroidism, diabetes, or the use of medications known to affect lipid metabolism.

### 2.4 Child assessments

All anthropometric and laboratory assessments were performed on the same day between 2023 and 2024, ensuring standardized collection conditions.

#### 2.4.1 Anthropometry

All anthropometric measurements were performed by trained nutritionists following standardized procedures and using calibrated equipment to ensure accuracy and reliability. Measurements were conducted according to the child's ability to stand, with specific procedures for children with and without microcephaly.

For children without microcephaly, weight was measured using a digital standing scale, with the child barefoot, wearing minimal clothing, standing upright with feet together and arms relaxed alongside the body. Height was measured using a stadiometer equipped with a non-stretchable 2-meter measuring tape, accurate to 0.1 cm, following World Health Organization (WHO) recommendations (24).

For children with microcephaly who were unable to stand independently, body weight was obtained using the traditional assisted weighing technique, in which the caregiver's weight is measured while holding the child, followed by subtraction of the caregiver's weight alone. This method is recognized as valid in field settings, particularly for populations with motor difficulties, and was applied by trained professionals using standardized protocols (2, 25). Recumbent length for children with microcephaly was measured using a portable infantometer, with the child lying on the measuring board, head against the fixed headboard, knees extended, feet together, and ankles at a 90° angle, secured by the movable footboard to ensure accuracy (25). For children able to stand, height was measured in the upright position using a stadiometer.

Body mass index (BMI) was calculated as weight (kg) divided by height squared (m²), using WHO Anthro Plus software (26). For the assessment of nutritional status, BMI-for-age Z-scores were calculated according to WHO 2007 growth standards. The Z-scores allow comparison of individual children's BMI with the reference population, taking age and sex into account. Waist circumference (WC) was measured with the child standing and arms uncrossed, at the midpoint between the lowest rib and the iliac crest, following the criteria described by Taylor et al. (27) Neck circumference (NC) was measured with the child standing, using a flexible measuring tape at the midpoint of the thyroid cartilage, perpendicular to the axis of the neck. WC and NC were included as part of the nutritional assessment to evaluate central adiposity.

#### 2.4.2 Lipid profile

After a 12-hour fast, 10 mL of venous blood was drawn from the antecubital vein into clot activator tubes, to analyze serum concentration of total cholesterol, high-density lipoprotein cholesterol (HDL-c), and triglycerides. Low-density lipoprotein cholesterol (LDL-c) was estimated using the Friedewald formula (28).

Lipid abnormalities were defined according to the First Brazilian Guideline for the Prevention of Atherosclerosis in Childhood and Adolescence (29, 47): total cholesterol <190 mg/dL; HDL-c > 40 mg/dL; triglycerides <150 mg/dL; LDL-c < 130 mg/dL. Atherogenic risk indices were calculated from the lipid profile data, as described by Kamoru et al. (30). The Castelli Risk Index I (CRI-I) was determined by the ratio of total cholesterol (CT) to HDL-c, while CRI-II corresponded to the ratio of LDL-c to HDL-c.

#### 2.4.3 Covariates

**Figure 1:**
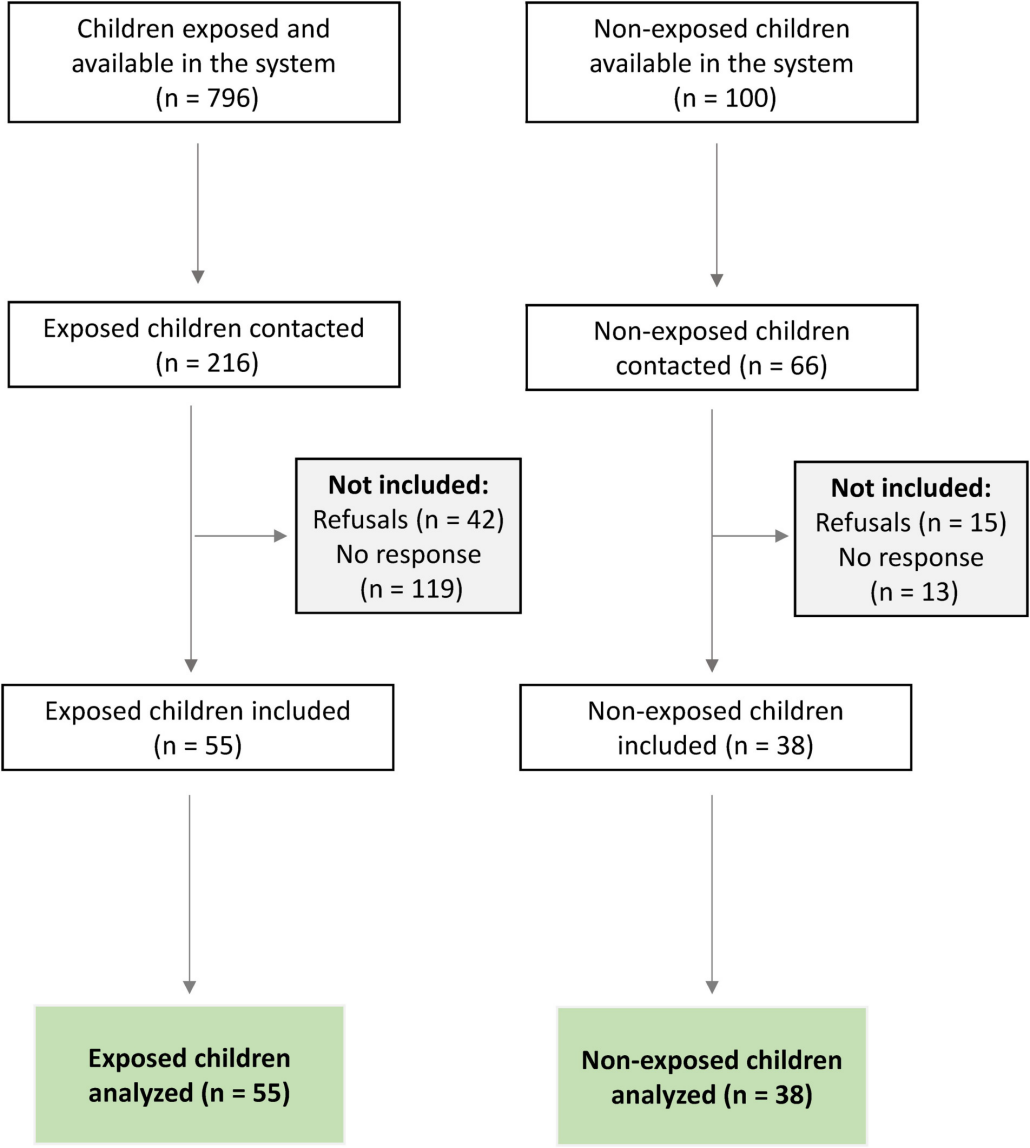
Flowchart of study participant selection. Source: Authors’ description of the participant selection process using data from SINAN and SINASC (2015–2017) and anthropometric measurements collected in 2023–2024.

Birth information was obtained from the child's and mother's health records. Additional data, such as skin color, family income, guardian's education level, child's screen time, and exclusive breastfeeding during the first six months of life, were collected through caregiver-administered questionnaires and structured interviews. Skin color, family income, and guardian's education level were self-reported by the caregivers, who selected the most appropriate option from predefined response categories. Screen time was assessed by caregivers, who reported the average daily hours the child spent watching television or using computers, tablets, or smartphones, and was categorized as ≤2 h/day or >2 h/day (Figure 1).

### 2.5 Data analysis

Data were entered in Microsoft Excel spreadsheets, and all statistical analyses were performed using STATA version 14 (StataCorp LP, College Station, TX, USA). A significance level was of 5% was adopted for all hypothesis tests. Categorical variables were expressed as absolute and relative frequencies, and Pearson's chi-squared test and Fisher's exact test were used to compare intrauterine ZIKV exposure, sociodemographic variables, screen time and breatfeeding up to 6 months with lipid profile alterations. Numerical variables were expressed as mean (standard deviation), and Student's *t*-test was performed to compare the means of anthropometric indicators according to intrauterine ZIKV exposure, sociodemographic variables, screen time and breatfeeding up to 6 months. Poisson regression models with robust variance were applied to assess the relationships between anthropometric variables and lipid profile alterations in children. Prevalence ratios (PR) with their respective 95% confidence intervals were calculated, both unadjusted and adjusted for sex, age, family income, screen time, and exclusive breastfeeding up to the sixth month. These covariates were selected based on the literature. The multiplicative interactions between anthropometric indicators and intrauterine ZIKV exposure were tested by including an interaction term in the adjusted Poisson regression model.

## 3 Results

Ninety-three children aged between 5 and 9 years were evaluated, with a mean (standard deviation—SD) of 6.5 (0.7) year-old, 58.1% (*n* = 54) were boys, and 59.1% (*n* = 55) had intrauterine exposed to the ZIKV. The overall mean (SD) values for lipid profile were: total cholesterol, 162.11 (30.32) mg/dL; HDL-c, 49.43 (11.62) mg/dL; LDL-c, 97.60 (26.39) mg/dL; and triglycerides: 75.45 (36.33) mg/dL. Anthropometric indicators showed mean (SD) BMI-for-age Z-score of −0.18 (1.84), waist circumference of 56.87 (8.48) cm, and neck circumference of 26.99 (2.22) cm.

Lipid alterations were higher in the study sample: 33.3% presented high total cholesterol, 44.1% low HDL-c, 26.9% high LDL-c, and 44.1% high triglycerides. A higher prevalence of low HDL-c levels was observed among children exposed to intrauterine ZIKV compared to unexposed ones (54.6% *vs.* 29.0%; *p* = 0.015). Regarding sociodemographic characteristics, children whose guardians had education above elementary school presented a higher prevalence of elevated triglycerides compared to those whose guardians had only completed elementary education (46.7% *vs.* 0.0%; *p* = 0.034). In addition, longer screen time (>2 h/day) was associated with a higher prevalence of low HDL-c (35.7% *vs.* 64.3%; *p* = 0.047) (Table 1).

**Table 1:** Lipid profile alterations according to intrauterine Zika virus exposure, sociodemographic characteristics, screen time, and breastfeeding up to 6 months.

Characteristics	*n* (%)	↑ Total cholesterol (yes)	↓ HDL-c (yes)	↑ LDL-c (yes)	↑ Triglycerides (yes)
All sample, *n* (%)		31 (33.3)	41 (44.1)	25 (26.9)	41 (44.1)
		*n* (%)	*n* (%)	*n* (%)	*n* (%)
Intrauterine Zika virus exposure					
Yes	55 (59.1)	17 (30.9)	30 (54.6)	14 (25.5)	27 (49.1)
No	38 (40.9)	14 (36.8)	11 (29.0)	11 (29.0)	14 (36.8)
*P*		0.551	**0.015** *	0.709	0.242
Sex					
Male	54 (58.1)	16 (29.6)	23 (42.6)	13 (24.1)	23 (42.6)
Female	39 (41.9)	15 (38.5)	18 (46.2)	12 (30.8)	18 (46.2)
*P*		0.373	0.733	0.472	0.733
Age					
5–6 years	39 (41.9)	17 (43.6)	19 (48.7)	14 (35.9)	16 (41.0)
7–9 years	54 (58.1)	14 (25.9)	22 (40.7)	11 (20.4)	25 (46.3)
*P*		0.075	0.445	0.096	0.613
Guardian's skin color					
White	9 (11.3)	1 (11.1)	5 (55.6)	1 (11.1)	4 (44.4)
Brown/black/yellow	71 (88.7)	23 (32.4)	31 (43.7)	18 (25.4)	30 (42.3)
*P*		0.266	0.724	0.678	0.999
Family income					
< 1 minimum wage	19 (24.1)	5 (26.3)	10 (52.6)	4 (21.1)	7 (36.8)
≥ 1 minimum wage	60 (75.9)	19 (31.7)	27 (45.0)	15 (25.0)	28 (46.7)
*P*		0.659	0.561	0.726	0.452
Guardian's education level					
Completed elementary school	6 (7.4)	1 (16.7)	2 (33.3)	0 (0.0)	0 (0.0)
Above elementary school	75 (95.6)	23 (30.7)	35 (46.7)	19 (25.3)	35 (46.7)
*P*		0.664	0.683	0.327	**0.034** **
Screen time					
≤ 2 h/d	14 (16.7)	4 (28.6)	9 (64.3)	4 (28.6)	7 (50.0)
> 2 h/d	70 (83.3)	27 (38.6)	25 (35.7)	21 (30.0)	28 (40.0)
*P*		0.557	**0.047** *	0.999	0.488
Exclusive breastfeeding up to 6 months					
Yes	33 (43.4)	11 (33.3)	14 (42.4)	8 (24.2)	16 (48.5)
No	43 (56.6)	11 (25.6)	21 (48.8)	9 (20.9)	18 (41.9)
*P*		0.460	0.578	0.731	0.565

Authors’ analysis of secondary data from the Brazilian national health information systems SINAN and SINASC, including children born during the ZIKV epidemic (2015–2017), and anthropometric data collected during the period 2023–2024. HDL-c, high-density lipoprotein cholesterol; LDL-c, low-density lipoprotein cholesterol.

The bold values indicate statistically significant results (*p* < 0.05).

* Pearson's chi-squared test, *p* < 0,05.

** Fisher's exact test, *p* < 0,05.

Anthropometric indicators did not differ significantly by exposure status. However, higher family income was associated with a higher BMI-for-age z-scores (*p* = 0.016). Older children (7–9 years) and those with guardians of Brown/Black/Yellow skin color presented higher mean neck circumference (*p* = 0.004 and *p* = 0.029, respectively) (Table 2).

**Table 2:** Anthropometric measurements according to intrauterine Zika virus exposure, sociodemographic characteristics, screen time, and breastfeeding up to 6 months.

Characteristics	BMI-for-age (z score)	Waist circumference (cm)	Neck circumference (cm)
	Mean (SD)	Mean (SD)	Mean (SD)
Intrauterine Zika virus exposure			
Yes	−0.41 (1.98)	55.9 (7.6)	27.3 (2.2)
No	0.15 (1.59)	58.1 (9.5)	26.6 (2.2)
*P*	0.147	0.224	0.106
Sex			
Male	−0.30 (1.74)	56.4 (8.0)	26.9 (2.1)
Female	−0.01 (1.99)	57.5 (9.1)	27.1 (2.4)
*P*	0.478	0.531	0.737
Age			
5–6 years	−0.38 (1.71)	55.2 (5.9)	26.2 (1.7)
7–9 years	−0.04 (1.93)	58.1 (9.8)	27.5 (2.4)
*P*	0.389	0.110	**0** **.** **004** *
Guardian's skin color			
White	−0.58 (1.66)	55.3 (7.0)	25.6 (1.4)
Brown/black/yellow	−0.01 (1.92)	57.9 (9.0)	27.4 (2.3)
*P*	0.399	0.419	**0** **.** **029** *
Family income			
< 1 minimum wage	−0.99 (2.38)	55.3 (9.5)	27.3 (2.1)
≥ 1 minimum wage	0.20 (1.63)	58.1 (8.4)	27.1 (2.3)
*P*	**0** **.** **016** *	0.227	0.824
Guardian's education level			
Completed elementary school	0.59 (0.52)	59.4 (6.6)	27.0 (1.6)
Above elementary school	−0.11 (1.94)	57.5 (8.9)	27.2 (2.3)
*P*	0.382	0.619	0.885
Screen time			
≤ 2 h/d	−0.42 (2.07)	53.9 (7.1)	26.4 (1.6)
> 2 h/d	−0.12 (1.69)	57.0 (8.6)	27.0 (2.1)
*P*	0.566	0.215	0.361
Exclusive breastfeeding up to 6 months			
Yes	−0.36 (1.97)	56.2 (8.5)	27.0 (2.3)
No	0.11 (1.81)	58.8 (8.9)	27.2 (2.3)
*P*	0.284	0.219	0.618

Authors’ analysis of secondary data from the Brazilian national health information systems SINAN and SINASC, including children born during the ZIKV epidemic (2015–2017), and anthropometric data collected during the period 2023–2024. BMI, body mass index; SD, standard deviation.

The bold values indicate statistically significant results (*p* < 0.05).

* Chi-squared test, *p* < 0,05.

**Fisher's exact test, *p* < 0,05.

In the regression models, BMI-for-age was inversely associated with low HDL-c (PR 0.87; 95% CI 0.78–0.97). In addition, significant interactions of intrauterine ZIKV exposure with BMI-for-age were observed in relationship with higher total cholesterol (*p* interaction = 0.005) and higher triglycerides (*p* interaction = 0.008). Among exposed children, BMI-for-age was strongly associated with increased total cholesterol (PR 1.96; 95% CI 1.43–2.69) (Table 3).

**Table 3:** Association between body mass index-for-age and lipid profile alterations in children, according to intrauterine Zika virus exposure.

Lipid profile	BMI-for-age (z score)			*p* interaction^2^
	All sample	Intrauterine Zika virus exposure		
		Yes	No	
	PR (95% CI)	PR (95% CI)	PR (95% CI)	
↑ Total cholesterol				
Unadjusted model	1.00 (0.87–1.15)	–	–	–
Adjusted model^1^	1.21 (0.99–1.48)	**1.96 (1.43–2.69)**	0.83 (0.54–1.26)	**0.005**
↓ HDL-c				
Unadjusted model	**0.89** (**0.80–0.98)**	–	–	–
Adjusted model^1^	**0.87** (**0.78–0.97)**	0.92 (0.83–1.03)	0.71 (0.41–1.22)	0.518
↑ LDL-c				
Unadjusted model	1.01 (0.86–1.19)	–	–	–
Adjusted model^1^	1.28 (0.99–1.65)	**1.68 (1.19–2.38)**	1.09 (0.68–1.75)	0.691
↑ Triglycerides				
Unadjusted model	0.95 (0.84–1.07)	–	–	–
Adjusted model^1^	0.99 (0.82–1.21)	0.89 (0.77–1.03)	1.36 (0.99–1.87)	**0.008**

Authors’ analysis of secondary data from the Brazilian national health information systems SINAN and SINASC, including children born during the ZIKV epidemic (2015–2017), and anthropometric data collected during the period 2023–2024. Bold values represent significant difference (*p* < 0.05) by Poisson regression with robust variance, *α* = 0.05. 95% CI: 95% confidence interval; BMI, body mass index; HDL-c, high-density lipoprotein cholesterol; LDL-c, low-density lipoprotein cholesterol; PR, prevalence ratio.

^1^ Model adjusted for child's sex, age, family income, screen time, and exclusive breastfeeding up to 6 months.

^2^ Multiplicative interaction between intrauterine Zika virus exposure and BMI-for-age (z score) in association with lipid profile alteration, adjusted for child's sex, age, family income, screen time, and exclusive breastfeeding up to 6 months.

There was a lack of association between waist circumference and lipid profile alterations in the adjusted model. However, a significant interaction between waist circumference and ZIKV exposure was observed for total cholesterol (p interaction = 0.029), with stronger associations among exposed children (PR 1.09; 95% CI 1.03–1.16) (Table 4).

**Table 4:** Association between waist circumference and lipid profile alterations in children, according to intrauterine Zika virus exposure.

Lipid profile	Waist circumference (cm)			*p* interaction^2^
	All sample	Intrauterine Zika virus exposure		
		Yes	No	
	PR (95% CI)	PR (95% CI)	PR (95% CI)	
↑ Total cholesterol				
Unadjusted model	0.98 (0.95–1.01)	–	–	
Adjusted model^1^	1.02 (0.98–1.06)	**1.09** **(1.03–1.16)**	0.97 (0.89–1.06)	**0**.**029**
↓ HDL-c				
Unadjusted model	0.99 (0.96–1.02)	–	–	–
Adjusted model^1^	0.97 (0.94–1.01)	0.99 (0.95–1.03)	0.93 (0.81–1.06)	0.565
↑ LDL-c				
Unadjusted model	0.98 (0.95–1.02)	–	–	–
Adjusted model^1^	1.03 (0.99–1.08)	**1.09** **(1.02–1.16)**	1.02 (0.94–1.10)	0.651
↑ Triglycerides				
Unadjusted model	1.01 (0.98–1.03)	–	–	–
Adjusted model^1^	1.02 (0.99–1.06)	0.99 (0.94–1.05)	**1.07** **(1.01–1.14)**	0.054

Authors’ analysis of secondary data from the Brazilian national health information systems SINAN and SINASC, including children born during the ZIKV epidemic (2015–2017), and anthropometric data collected during the period 2023–2024. Bold values represent significant difference (*p* < 0.05) by Poisson regression with robust variance, *α* = 0.05. 95% CI: 95% confidence interval; BMI, body mass index; HDL-c, high-density lipoprotein cholesterol; LDL-c, low-density lipoprotein cholesterol; PR, prevalence ratio.

^1^ Model adjusted for child's sex, age, family income, screen time, and exclusive breastfeeding up to 6 months.

^2^ Multiplicative interaction between intrauterine Zika virus exposure and waist circumference (cm) in association with lipid profile alteration, adjusted for child's sex, age, family income, screen time, and exclusive breastfeeding up to 6 months.

Neck circumference was positively associated with total cholesterol (PR 1.22; 95% CI 1.02–1.46), LDL-c (PR 1.33; 95% CI 1.02–1.73), and triglycerides (PR 1.20; 95% CI 1.03–1.39). The magnitude of the association was greater in the exposed group, although interaction terms were not statistically significant (Table 5).

**Table 5:** Association between neck circumference and lipid profile alterations in children, according to intrauterine Zika virus exposure.

Lipid profile	Neck circumference (cm)			*p* interaction^2^
	All sample	Intrauterine Zika virus exposure		
		Yes	No	
	PR (95% CI)	PR (95% CI)	PR (95% CI)	
↑ Total cholesterol				
Unadjusted model	0.93 (0.82–1.07)	–	–	
Adjusted model^1^	**1.22** (**1.02–1.46)**	**1.44** (**1.08–1.93)**	1.09 (0.83–1.43)	0.304
↓ HDL-c				
Unadjusted model	0.97 (0.86–1.11)	–	–	–
Adjusted model^1^	0.93 (0.77–1.14)	0.96 (0.77–1.20)	**0.60** (**0.39–0.94)**	0.191
↑ LDL-c				
Unadjusted model	0.96 (0.82–1.12)	–	–	–
Adjusted model^1^	**1.33** (**1.02–1.73)**	**1.64** (**1.09–2.48)**	1.17 (0.84–1.61)	0.336
↑ Triglycerides				
Unadjusted model	1.04 (0.94–1.16)	–	–	–
Adjusted model^1^	**1.20** (**1.03–1.39)**	**1.37** (**1.04–1.80)**	1.09 (0.84–1.42)	0.384

Authors’ analysis of secondary data from the Brazilian national health information systems SINAN and SINASC, including children born during the ZIKV epidemic (2015–2017), and anthropometric data collected during the period 2023–2024. Bold values represent significant difference (*p* < 0.05) by Poisson regression with robust variance, *α* = 0.05. 95% CI: 95% confidence interval; BMI, body mass index; HDL-c, high-density lipoprotein cholesterol; LDL-c, low-density lipoprotein cholesterol; PR:,revalence ratio.

^1^ Model adjusted for child's sex, age, family income, screen time, and exclusive breastfeeding up to 6 months.

^2^ Multiplicative interaction between intrauterine Zika virus exposure and neck circumference (cm) in association with lipid profile alteration, adjusted for child's sex, age, family income, screen time, and exclusive breastfeeding up to 6 months.

Castelli Index I was significantly higher in the exposed group (3.53 ± 0.89^a^) than in the non-exposed group (3.15 ± 0.81^a^), as indicated by the (p interaction = 0.0389). In contrast, no statistically significant difference was observed for Castelli Index II, with values of (2.16 ± 0.75^a^) in the exposed group and (1.93 ± 0.57^a^) in the non-exposed group (p interaction = 0.1087).

## 4 Discussion

This study investigated the associations between intrauterine exposure to the ZIKV and anthropometric indicators with lipid profile alterations in school-aged children born during the Zika epidemic in Brazil. The main findings were: (1) a higher prevalence of low HDL-c among children exposed to ZIKV *in utero* compared to unexposed peers; (2) significant interactions between ZIKV exposure and adiposity indicators (BMI-for-age and waist circumference) for total cholesterol and triglycerides; and (3) positive associations of neck circumference with total cholesterol, LDL-c, and triglycerides among all children; and (4) higher Castelli I index values compared to unexposed peers, whereas the Castelli II index exhibited similar patterns between groups. These results suggest that metabolic changes may occur early in life among ZIKV-exposed children, even in the absence of overt anthropometric differences.

Children exposed to ZIKV *in utero* showed a significantly higher prevalence of low HDL-c, suggesting that gestational exposure may influence lipid metabolism. This aligns with growing evidence that congenital viral infections can alter metabolic programming via inflammatory, endocrine, and mitochondrial pathways that persist beyond birth (29, 31). Experimental studies indicate that ZIKV infection affects lipid homeostasis in neural and hepatic tissues, increasing oxidative stress and disrupting cholesterol efflux mechanisms (32, 33). Such disturbances in early lipid regulation may contribute to long-term cardiometabolic risk, supporting the hypothesis that ZIKV exposure during fetal life exerts enduring systemic effects beyond the well-documented neurodevelopmental outcomes.

Our results show that Castelli I index, which represents the ratio of total cholesterol to HDL-c and is a sensitive marker of overall lipid balance, was higher in children with intrauterine exposure to ZIKV, suggesting greater metabolic vulnerability even in the absence of significant differences in other lipid parameters. On the other hand, Castelli II index, which represents the LDL-c/HDL-c ratio and is considered a more direct atherogenic marker, did not differ significantly between groups. Although no published studies have directly assessed Castelli indices in children with prenatal ZIKV exposure, experimental and lipidomic findings suggest that maternal infection can reprogram fetal lipid metabolism. Studies in placenta and neural cells have shown lipid accumulation, alterations in lipogenesis, and increased oxidative stress (33, 34, 48), while lipidomic analyses in exposed newborns reveal changes in serum lipid species (35). These findings provide original evidence regarding cardiometabolic risk in this population, indicating that prenatal ZIKV exposure may exert subtle yet detectable effects on the overall balance of lipid fractions, filling an important gap in the literature.

A novel aspect of this study is the identification of significant interactions between ZIKV exposure and adiposity indicators. Among exposed children, higher BMI-for-age was strongly associated with increased total cholesterol and triglycerides. Similarly, waist circumference showed a stronger positive association with total cholesterol among exposed participants. These results suggest that the combination of adverse intrauterine programming and postnatal adiposity may amplify cardiometabolic risk (3, 36–39). Comparable findings have been reported for other congenital infections, such as (3, 40). where early inflammatory activation modulates adipocyte function and lipid synthesis (32, 41). In this context, children prenatally exposed to ZIKV may exhibit increased metabolic sensitivity to weight gain, reinforcing the importance of early lifestyle interventions in this group.

Considering the entire sample, BMI-for-age was inversely associated with low HDL-c, which may reflect the compensatory role of adiposity in maintaining HDL-c levels within certain physiological limits in early childhood (42). In younger children, moderate increases in body fat can transiently elevate HDL-c concentrations due to enhanced hepatic synthesis of apolipoprotein A-I and shifts in lipoprotein particle size distribution (30). However, this apparent “protective” association may not persist with advancing age, when sustained adiposity tends to promote pro-inflammatory states and HDL-c decline.

Conversely, neck circumference was positively associated with total cholesterol, LDL-c, and triglycerides, confirming its utility as a simple and reliable anthropometric marker of central adiposity and lipid imbalance (43). Cervical fat deposition reflects early visceral fat expansion and is metabolically active, contributing to systemic inflammation, altered lipid transport, and insulin resistance (39, 44). The greater magnitude of these associations among ZIKV-exposed children, although not statistically significant in interaction terms, suggests that prenatal viral exposure may potentiate the metabolic consequences of central fat accumulation, possibly through persistent mitochondrial or inflammatory dysregulation (39, 43–46). These findings underscore the importance of neck circumference alongside traditional anthropometric measures in metabolic risk screening, particularly in populations exposed to intrauterine infections.

Behavioral and socioeconomic variables were also associated with lipid alterations. Longer screen time (>2 h/day) was associated with a higher prevalence of low HDL-c, corroborating previous studies linking sedentary behavior and prolonged exposure to electronic media with reduced energy expenditure, altered lipoprotein metabolism, and increased cardiometabolic risk in children (29, 31). Unexpectedly, children whose caregivers had education beyond elementary school presented higher triglyceride levels. This paradox likely reflects the nutritional transition occurring in middle-income countries, where higher education and income do not always translate into healthier lifestyles. Increased purchasing power and urban living can lead to greater reliance on processed foods, reduced physical activity, and exposure to obesogenic environments shaped by aggressive food marketing and time scarcity (29, 31). Moreover, higher family income was associated with higher BMI-for-age Z-scores, suggesting that socioeconomic advancement may coexist with behaviors that promote excess adiposity. These findings illustrate the complex, non-linear relationship between socioeconomic position and metabolic health in transitional contexts.

This study makes novel and important contributions to understanding the metabolic consequences of intrauterine ZIKV exposure. The retrospective cohort design establishes temporality between gestational exposure and childhood metabolic outcomes, providing an improvement over cross-sectional studies and including an appropriate control group. The use of multiple anthropometric markers—BMI, waist circumference, and neck circumference—enhances the assessment of childhood adiposity and identification of distinct fat distribution patterns. Rigorous statistical control for potential confounders, including age, sex, family income, screen time, and exclusive breastfeeding, further strengthens the internal validity of the findings. The demonstration of ZIKV exposure–adiposity interactions contributes novel insights into how prenatal viral infection and postnatal growth jointly shape metabolic risk.

This study has some limitations. The use of a non-probabilistic sample may limit the generalizability of the findings. Although the analyses were adjusted for relevant covariates, data on physical activity, dietary intake, and inflammatory markers were not available, precluding a more comprehensive assessment of the mechanisms underlying the observed dyslipidemias. In addition, confirmation of ZIKV exposure relied on secondary records, which may be subject to underreporting or diagnostic inconsistencies. Importantly, no additional clinical conditions known to affect lipid metabolism were identified among children with microcephaly, reducing the likelihood of confounding by underlying metabolic disorders.

Maternal information was carefully considered; however, the available obstetric clinical history variable included only diabetes mellitus and hypertension and did not specifically capture maternal chronic degenerative diseases. Moreover, this variable presented a high proportion of missing data (>10%), which substantially reduced the effective sample size and statistical power in exploratory analyses. Therefore, it was not included in the final models.

## 5 Conclusions

Interactions between intrauterine ZIKV exposure and adiposity indicators (BMI-for-age and waist circumference) were associated with higher total cholesterol and triglyceride levels, suggesting that adiposity may have a more pronounced metabolic impact in exposed children. Castelli indices indicated cardiovascular risk, with CI-I significantly higher in the exposed group, reflecting a global imbalance in lipid fractions and early metabolic vulnerability. Neck circumference also emerged as a reliable marker, positively associated with total cholesterol, LDL-c, and triglycerides. These findings support the use of multiple anthropometric measures, alongside consideration of behavioral and socioeconomic factors, for the early identification of children at risk of dyslipidemia, particularly those prenatally exposed to ZIKV.

## Data Availability

The raw data supporting the conclusions of this article will be made available by the authors, without undue reservation.
